# Polymorphism and the Red Queen: the selective maintenance of allelic variation in a deteriorating environment

**DOI:** 10.1093/g3journal/jkae107

**Published:** 2024-05-21

**Authors:** Hamish G Spencer, Callum B Walter

**Affiliations:** Department of Zoology, University of Otago, Dunedin 9054, New Zealand; Department of Zoology, University of Otago, Dunedin 9054, New Zealand

**Keywords:** natural selection, genetic variation, mathematical model, constructionist model, single-locus model

## Abstract

Although allelic variation is ubiquitous in natural populations, our theoretical models are poor at predicting the existence and properties of these observed polymorphisms. In this study, inspired by Van Valen's Red Queen hypothesis, we modeled the effect of viability selection in a deteriorating environment on the properties of allelic variation in populations subject to recurrent mutation. In Monte Carlo simulations, we found that levels of polymorphism consistently built up over time. We censused the simulated populations after 10,000 generations of mutation and selection, revealing that, compared with models assuming a constant environment, the mean number of alleles was greater, as was the range of allele numbers. These results were qualitatively robust to the addition of genetic drift and to the relaxation of the assumption that the viabilities of phenogenotypes containing a new mutation are independent of each other (i.e. incorporating a model of generalized dominance). The broad range of allele numbers realized in the simulated populations—from monomorphisms to highly polymorphic populations—more closely corresponds to the observed range from numerous surveys of natural populations than previously found in theoretical studies. This match suggests that, contrary to the views of some writers, selection may actively maintain genetic variation in natural populations, particularly if the selective environment is gradually becoming harsher. Our simulations also generated many populations with heterozygote advantage, a mismatch with real data that implies that this selective property must arise extremely rarely in natural populations.

## Introduction

In his agenda-setting critique of population genetics, Harvard geneticist Richard Lewontin (1974) argued that the central problem in the field was the mismatch between observations of high levels of polymorphism and the theories proposed to explain that variation. Much progress has been made in the intervening 50 years with, for example, the development of the nearly neutral theory (Kimura 1984). Similarly, models of selective sweeps have shown how levels of variation at and nearby a genetic locus can be shaped by the fixation of advantageous mutations (Schrider and Kern 2017). Nevertheless, the paradox pointed out by Lewontin remains (Lewontin 1974; Kern and Hahn 2018; Buffalo 2021).

Arguments that selection is largely responsible for the maintenance of genetic variation derive from the single-locus, 2-allele model of heterozygote advantage in an infinite population. The globally stable equilibrium of this basic model maintains both alleles indefinitely. The extension of these ideas to more alleles, however, has proved problematic. Heterozygote advantage (whether “pairwise” or “total”; see Lewontin *et al*. 1978) is neither necessary nor sufficient to maintain more than 2 alleles.

Worse, as Lewontin *et al*. (1978) showed, the fitness parameters that maintained larger numbers of alleles in the standard model of viability selection were extremely unusual. For a fixed number of alleles, they used computer simulations to generate random sets of viabilities. For each set, they found the equilibria of the equations describing the changes in allele frequencies, which they tested for feasibility (i.e. having nonnegative allele frequencies) and stability (so that all the alleles would be maintained). The proportion of the random fitness sets that maintained all alleles rapidly declined as the number of alleles increased, just 6 of 10^5^ sets for 5 alleles and none for 6 alleles. The same result obtained in other models of selection: sexually differential (Marks and Ptak 2001), frequency-dependent (Trotter and Spencer 2007), spatially heterogenous (Star *et al.* 2007a), and constant fertility (Clark and Feldman 1986).

These findings—that the proportion of random fitness sets affording stable polymorphism is extremely small for larger numbers of alleles—were widely held to be evidence against selective explanations for the maintenance of variation (e.g. Árnason 1982; Kimura 1984; Keith *et al*. 1985; Nevo 2001; Charlesworth 2006; Gloria-Soria *et al*. 2012; Ejsmond *et al*. 2014). Such interpretations, however, are logically flawed: quite generally, the size of some part of parameter space affords almost no inferences about the likelihood of systems being there. Indeed, selection is known to seek out very unusual outcomes, both phenotypic and genotypic.

Incorporating a historical dimension to these models, by adding recurrent mutation over time, paints a very different picture. In what has become known as the “constructionist” approach (Taylor 2005), the focus shifts from the equilibrium of the system to the evolution of the system over time. Constructionist approaches are part of a wider recognition of the value of explicitly incorporating historical dimensions into eco-evolutionary research (Spencer 2020). In population genetics, for example, a typical constructionist model of selection (e.g. Spencer and Marks 1988, 1992; Marks and Spencer 1991) examines how the number of alleles changes over time as, each generation, one or more novel mutants are introduced to the existing set of alleles before selection acts to mold their frequencies. Most often, of course, selection eliminates the new mutation and no change occurs. Occasionally, however, a mutant successfully invades the population and, on other occasions, an existing allele is driven to extinction. Thus, the number of alleles (and all the other properties of the system) evolves over time. The population is then censused at some arbitrary time point (e.g. after 10,000 generations), which gives a measure of how easily polymorphisms with particular numbers of alleles are constructed. Different models examine various forms of selection and methods for generating fitnesses. A not dissimilar approach was used by Kisdi and Geritz (1999) to examine the evolution of polymorphism in a heterogeneous environment via mutations of small effect. In all these models, populations evolve so that polymorphism is actively maintained; the small size of the parts of parameter space affording polymorphism is not a long-term barrier to selection preserving alleles with such parameters.

Using the exact same model of selection used by Lewontin *et al*. (1978), but with novel mutations added each generation, Spencer and Marks (1988, 1992) and Marks and Spencer (1991) showed that systems with higher levels of polymorphism were easily constructed overtime: although most mutations were immediately eliminated by selection, very occasionally, a mutant would be able to invade the population, and polymorphism built up over time. Again, these first results extended to other models of selection (Marks and Ptak 2001; Star *et al*. 2007b, 2008; Trotter and Spencer 2008, 2013).

Nevertheless, the modeling to date has struggled to predict the full range of genetic variation observed in natural populations. Models generating higher levels of polymorphism were unable to produce monomorphisms (e.g. Spencer and Marks 1992) and vice versa (e.g. Spencer and Mitchell 2016). Improving the realism of the models, for example, with the addition of genetic drift (Star and Spencer 2013), only partially solved the issue.

In this study, we examine a heuristically important modification to the standard model of constant viability selection, the Red Queen model (Van Valen 1973). In this influential hypothesis, the fitness of the species of interest declines over time as the other biological components of the environment (e.g. competitors, predators, prey, or parasites) adapt to its presence (Brockhurst *et al*. 2014; Strotz *et al*. 2018). Nevertheless, abiotic change may also act as the agent for gradual fitness decline, for example, as various physical resources are depleted or polluted. Whatever the source of environmental degradation, the fitness of the species of interest will decrease in the absence of novel genetic variation. Like the Red Queen in *Alice Through the Looking Glass*, therefore, the species must constantly “run” (i.e. improve its fitnesses) to simply stay where it is.

Biotic interactions will often result in coevolutionary changes: at the same time, as the species of interest is adapting in response to the selection pressures induced by the presence of the other species in the environment, the latter are themselves adaptively changing in response to their interactions with the first species. Intuitively, a deteriorating selective environment might be expected to result in larger numbers of alleles than a constant environment, as new, fitter alleles enter the population, replacing those that are older and less fit.

Explicitly coevolutionary models (which we do not study here) often invoke negative frequency-dependent selection, in which rarer phenogenotypes have a selective advantage. Again, such a selective regime might be expected to maintain higher levels of genetic variation. But recent work has shown that such expectations need not be fulfilled. For example, ecological and epidemiological feedbacks in models of negative frequency-dependent selection arising from antagonistic coevolution between hosts and their parasites can sometimes hinder the maintenance of genetic variation (MacPherson *et al*. 2021).

Our goal was to follow the evolution of theoretical polymorphisms constructed over time, via recurrent mutation and selection, under the assumptions of a deteriorating environment. We implemented computer simulations using the constructionist approach to examine the properties of the resultant polymorphisms: number of alleles, mean fitnesses, allele frequencies, etc.

## Model

We start with the standard model of viability selection at a single locus with *n* alleles in an infinite diploid population with discrete generations, so that the frequency of allele *A_i_* iterates according to


(1) $$\bar{w}{\;p}_{i}^{\prime }=p_{i}\overset{n}{\underset{\;j=\;1}{\sum }}\;w_{ij}p_{j}$$

in which *p_i_* is the frequency of allele *A_i_*, *w_ij_* (= *w_ji_*) is the viability of the phenogenotype *A_i_A_j_*, and $\bar{w}$ is the mean fitness of the population given by the sum over *i* of the right-hand side of the equation. We studied both infinite populations and those with finite size *N* ∈ {10^3^, 10^4^, 10^5^, 10^6^}.

In order to do so, we wrote a novel computer program that started with a single allele (*n* = 1) with a fitness, *w*_11_. Each generation, we created a novel mutation, *A_n_*  _+ 1_, which had an initial frequency of 5 × 10^−5^ (infinite populations) or 1/2*N* (finite populations). We also generated the associated *n* + 1 initial viability parameters for the possible new phenogenotypes, using 1 of the 2 methods. First, in keeping with Spencer and Marks (1988), these values were chosen independently from the unit interval [0, 1]. We started these simulations with *w*_11_ = 0.5. This approach allows us to make a direct comparison with the results of Spencer and Marks (1988) and evaluate the direct effects of the deteriorating environment of the Red Queen.

Second, in order to improve the realism of our simulations, we generated the initial viabilities according to the “generalized dominance” method employed by Spencer and Mitchell (2016). Generalized dominance assumes that there is a major allelic effect on viabilities, a view motivated by the fact that gene products are usually translated from a single allele. To do so, we generated a primary effect, *X_n_*  _+ 1_, together with *n* + 1 interaction effects, *Y_i_*_, *n*_  _+ 1_ (*i* = 1, 2, …, *n* + 1), independently from the uniform distribution on [0, 1]. Then,


(2) $$w_{ij}\;=\alpha (X_{i}+X_{j})+(1-2\alpha )Y_{ij}$$

in which the weight, *α*, was a fixed constant between 0 and ½ (usually ⅓ in our runs). In these runs, the initial fitness *w*_11_ was not ½ but $2\alpha X_{1}+(1-2\alpha )Y_{11}$. When *α* = 0, the *w_ij_* are independent, and the 2 methods are identical. In general, the correlation between the viabilities of genotypes sharing an allele, *A_i_*, is


(3) $$Cor(w_{ij},w_{ik})=\frac{\alpha^{2}}{1-4\alpha +6\alpha^{2}}$$

When *α* = ⅓, this correlation is ⅓.

Following the generation of the new mutant, we iterated allele frequencies according to Equation (1). A new mutation, *A_n_*  _+ 1_, will invade an infinite population when its marginal fitness is greater than the population's mean fitness (i.e. $\sum_{\;j=\;1}^{n+\;1}w_{\;jn+\;1}p_{j}>\bar{w}$).

In versions of the model with genetic drift, we took a sample of size 2*N* with replacement (a multinomial sampling process with the allele frequencies as the probabilities), thus producing the next generation's allele frequencies. At this point, we checked for the extinction of alleles, removing them from the system. In finite populations, extinction occurred when an allele's frequency after genetic drift fell to zero. In the infinite-population simulations, we rounded any frequency below 5 × 10^−5^ down to zero.

We incorporated the deteriorating fitnesses of the Red Queen into our simulations in 1 of the 2 ways. First, we assumed a constant multiplicative decrease in fitnesses, *d* ∈ {0.995, 0.999, 0.9999}, each generation, so that


(4) $$w_{ij}^{\prime }=dw_{ij}$$

We also used *d* = 1 to replicate the results of previous workers using the constant viability model. In the simulations of generalized dominance, we also reduced the primary effects for each allele in each generation in the same way:


(5) $$X_{i}^{\prime }=dX_{i}$$

In a second series of simulations, the decay in fitness was a random variable, *d_ij_*, sampled from a normal distribution, Norm(*µ*, *σ*^2^), with 15.87% of its values >1 [e.g. Norm(0.999, 0.001^2^)]:


(6) $$w_{ij}^{\prime }=d_{ij}w_{ij}$$

Pseudorandom numbers were provided by the lagged-Fibonacci generator of Marsaglia *et al*. (1990). Simulations were run for 10^4^ generations, at which point we recorded frequencies and viabilities for those alleles still extant for later analysis. In addition, we followed a small number of exemplar simulations in detail, recording allele numbers and mean fitnesses every generation. We considered alleles at frequencies >0.01 to be common.

We measured the location of a polymorphism in the state space of possible common-allele frequencies after 10^4^ generations with the square of the Euclidean distance to the centroid:


(7) $$I=\;\overset{n_{c}}{\underset{c=\;1}{\sum }}\;{\left(\;p_{c}-\frac{1}{n_{c}}\right)}^{2}$$

where the index *c* runs over the range of common alleles. We excluded rare alleles in this calculation, as they bias the location to the edges of state space but are not an important element of the polymorphism. Approximately equal (common) allele frequencies give small values of *I*; uneven ones give larger ones.

We compared our empirical distributions of *I* values with the null expectation of *I* calculated from random allele frequencies. These latter values were generated using the broken-stick method (Holst 1980), which selects random *n_c_* − 1 break points on the unit interval, [0, 1], the allele frequencies being the differences between adjacent break points. This process ensures that our null hypothesis *I* values correspond to uniformly distributed allele frequencies in the (*n_c_* − 1)-dimensional simplex of possible values,


$$\left\{(p_{1},p_{2},\ldots ,p_{n_{c}}):\;0<p_{i}\;\mathrm{for}\;\mathrm{all}\;i\;\mathrm{and}\;\sum_{i}^{n_{c}}\;p_{i}=1\right\}$$

The Delphi codes implementing these simulations are summarized in the Supplementary Files 1–9. Separate programs were used to generate the data for different versions of these simulations; descriptions are given in the Supplementary Material.

## Results and analysis

### Temporal trajectories

**Fig. 1.**
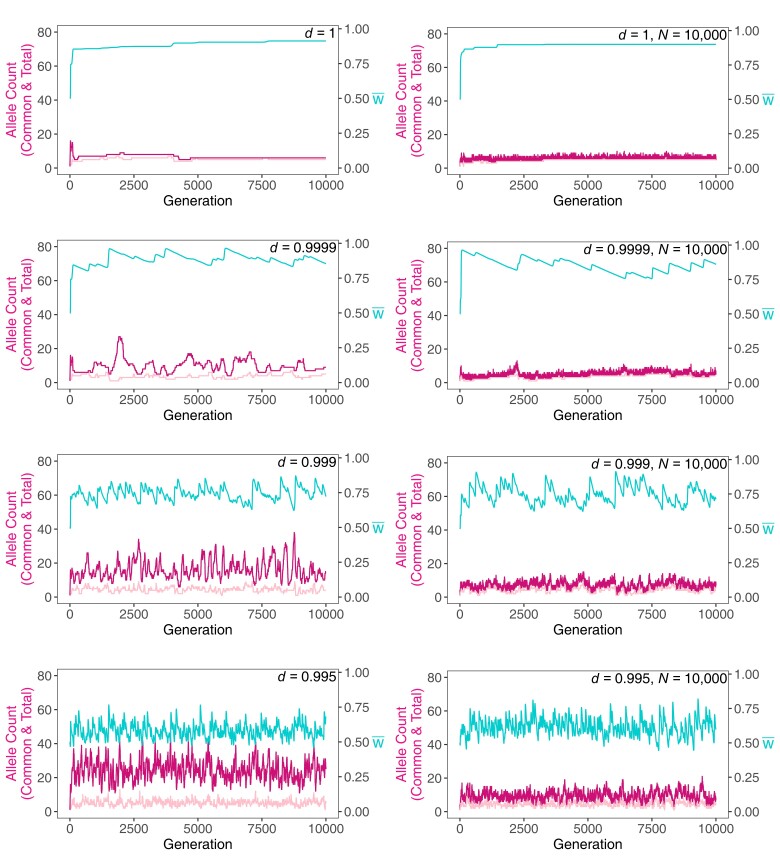
Number of common alleles (lowest line in pale pink; *n_c_*), total number of alleles (middle line in magenta; *n*), and mean fitness (uppermost line in blue; $\bar{w}$ ) over time in simulated populations without genetic drift (left column) and with drift (population size *N* = 10,000, right column), for different environmental decay rates, *d*, as shown in each panel.

The evolution of some exemplar populations is shown in Fig. 1. In the infinite population model with *d* = 1 (i.e. the standard model), we see that, as Kingman (1961) proved, the mean fitness increases over time. Even when we incorporate drift (*N* = 10^4^), this property holds almost always, although drift-induced extinction dramatically increases the turnover of alleles. Most alleles were common.

Turning to our Red Queen simulations, we can see immediately (Fig. 1) that increasing the rate of decay (smaller values of *d*) results in a greater turnover of alleles, as well as larger numbers of alleles. This effect arises from the greater invasion probability due to a given new mutation having a fixed marginal fitness more likely to be greater than the faster declining mean fitness.

It is worth noting that almost never did an allele reach fixation at any stage. None of the simulations in Fig. 1 reduced to *n* = 1 (or even *n_c_* = 1). Although every allele is eventually headed for extinction and thus could be viewed as transient (as in the neutral hypothesis), a large part of the allelic variation is likely to be actively maintained by selection: The population would reach a polymorphic equilibrium with those alleles if the environment ceased deteriorating. At any given time point, very little of the standing variation is already headed to extinction.

The trajectory of the mean fitness over time is strongly affected. There is a pattern (particularly evident for moderate decay, *d* = 0.9999) of declines over longer periods, followed by rapid increases as several advantageous new mutations invade. In other words, when *d* < 1, the tendency for selection to increase mean fitness (which occurs every generation when *d* = 1) is episodic. The invasion of new mutants (with greater fitnesses) does not immediately compensate for the decline in mean fitness induced by decay.

Comparing populations with and without drift shows (see also Supplementary Fig. 1), as expected, that drift reduces allele numbers and increases allelic turnover. Some alleles that are advantageous (and thus would be expected to invade in an infinite population) drift to extinction. The number of common alleles fluctuates much less than does the total number in all cases.

Simulations with a variable decay parameter showed similar results (see Supplementary Fig. 2), but the total number of alleles was lower. Generalized dominance also gave comparable patterns, although the total number of alleles was slightly higher (Supplementary Fig. 2).

### Numbers of alleles

**Fig. 2.**
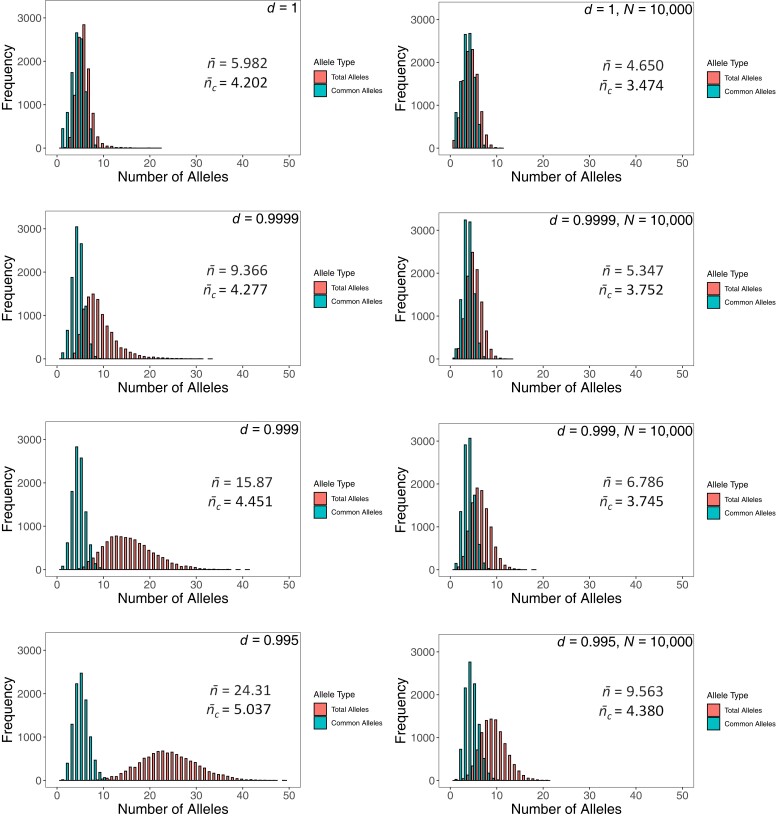
Frequency distributions of the numbers of common (*n_c_*) and total (*n*) alleles at generation 10,000 in 10^4^ simulations without drift (left-hand column) and with drift (population size *N* = 10,000, right column). The means of each distribution are shown in each panel.

We recorded the total and common numbers of alleles from a large number (10^4^) of replicate simulations (differing only in their pseudorandom-number seeds) at generation 10,000. Bar charts of the frequencies of these numbers are shown in Fig. 2, confirming the single-run observations detailed above. Greater decay leads to more total and common alleles, although the change in the latter is small (see also Supplementary Fig. 3). Again, this trend arises because decaying fitnesses allow more successful invasions and lead to the more rapid selective elimination of existing alleles.

The addition of genetic drift reduces the total and common allele numbers, but the effect is far greater for the total number, especially with a smaller *d* (see Fig. 2 and Supplementary Fig. 4). Smaller populations end up with fewer common and, especially, total alleles (Supplementary Fig. 3).

Once again, the results with variable *d* and generalized dominance (with or without drift) gave very similar distributions (Supplementary Figs. 5 and 6), although the means of these distributions were usually somewhat smaller.

### Mean fitnesses

**Fig. 3.**
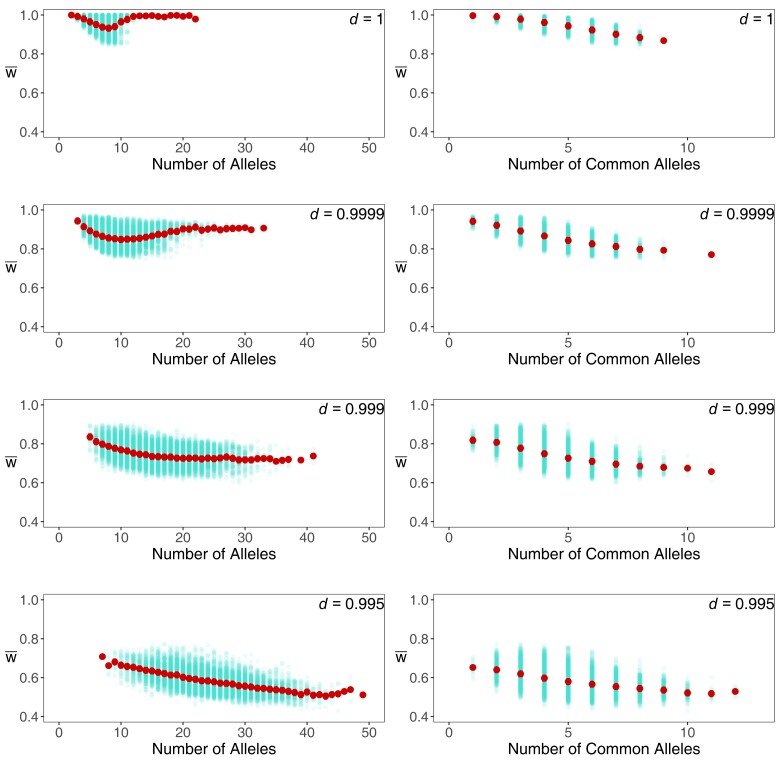
Distributions of the mean fitness ($\bar{w}$ ) at generation 10,000 in 10^4^ simulations without drift, for the final total numbers of alleles (*n*; left-hand column) and common alleles (*n_c_*; right-hand column). The means of each distribution are shown by the large red dots in red.

Figure 3 shows the mean and distribution of fitnesses for populations at generation 10,000, binned by the number of alleles (*n*, on the left) or common alleles (*n_c_*, on the right). This binning is necessary because populations with more alleles have, of course, more fitness parameters, which are constrained to be between 0 and 1. Hence, any weighted average (such as the mean fitness) is likely to be smaller. Note that for some values of *n*, the sample mean shown is affected by small sample sizes.

Faster decay (smaller *d*) consistently led, in the nondrift simulations, to lower mean fitnesses for all numbers of alleles, total and common (Fig. 3). As expected, populations with more alleles (both common and total) generally exhibited a lower mean fitness, in line with previous findings (e.g. Spencer and Chiew 2015; Spencer and Mitchell 2016). For a slower decay, however, mean fitness was the lowest for intermediate values of *n* (but not for *n_c_*). This result is an artifact of the presence, in populations with greater *n*, of rare alleles on their way to extinction that do not greatly affect $\bar{w}$, which is thus higher than expected. Indeed, for a small number of runs with *d* = 1, $\bar{w}$ is close to 1.0 for a larger *n*: presumably, these populations possess a recent mutation with high fitnesses and close to fixation, as well as several rare alleles. It is noteworthy that we do not see this phenomenon when examining only common alleles.

The effect of drift is small: compared with populations without drift, finite populations generally have lower mean fitnesses across all decay rates, for both *n* and *n_c_* (Supplementary Fig. 7). Differences among finite populations of different sizes were negligible, however.

### Fitness structures

**Fig. 4.**
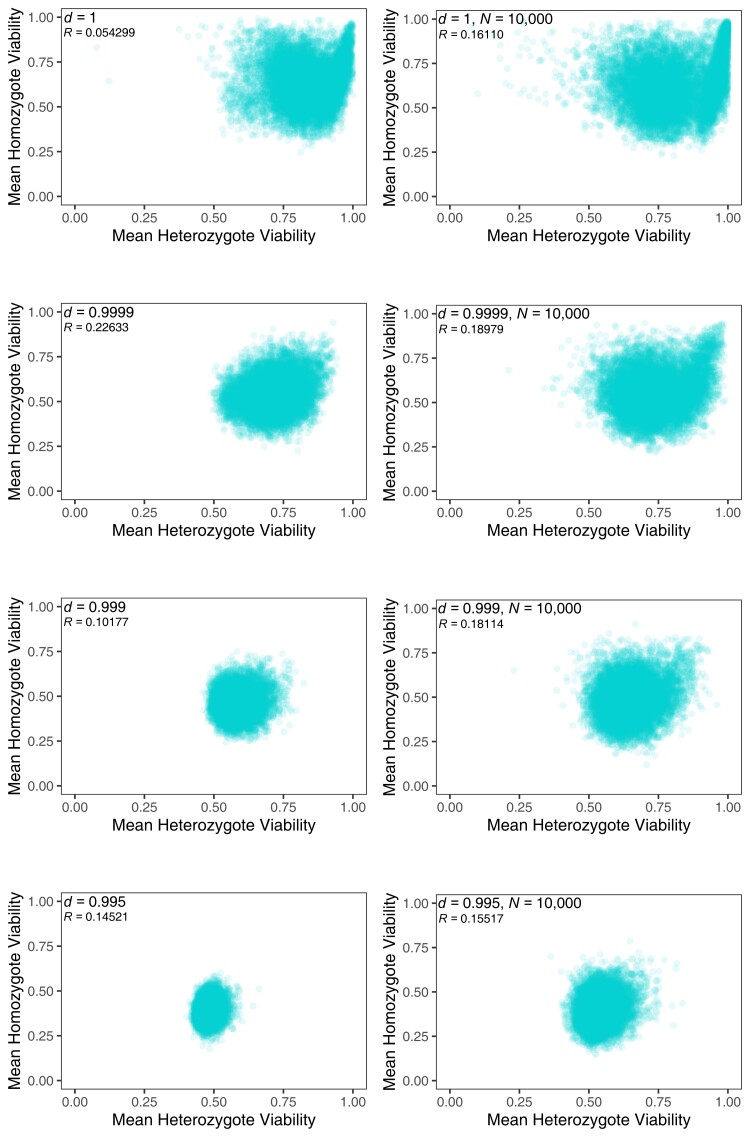
Scatterplots of the mean of the homozygote viabilities vs the mean of the heterozygote viabilities for 10^4^ simulations each at generation 10,000, without drift (left-hand column) and with drift (*N* = 10,000; right-hand column), for different decay rates as shown in the panels. Pearson's correlation values are shown in each panel.

We examined the structure of the sets of fitnesses for alleles extant at generation 10,000. The within-population means of heterozygote viabilities are always positively but weakly correlated with the means of their homozygote fitnesses (Fig. 4). The correlation is much lower in infinite populations with no fitness decay, however, because fitnesses, both homozygous and heterozygous, accumulate near the upper boundary of 1.0. Introducing decay means fitnesses decline away from 1.0, which leads to stronger correlations.

Faster decay also led to both these means being smaller on average, and the range of values was also less. Simulations incorporating drift showed that smaller populations had a greater range of mean heterozygote and homozygote fitnesses for all decay rates. The smallest populations (*N* = 10,000) had slightly higher heterozygote means on average than larger and infinite populations, as well as a greater range (Fig. 4 and Supplementary Fig. 8).

### Location of polymorphisms

**Fig. 5.**
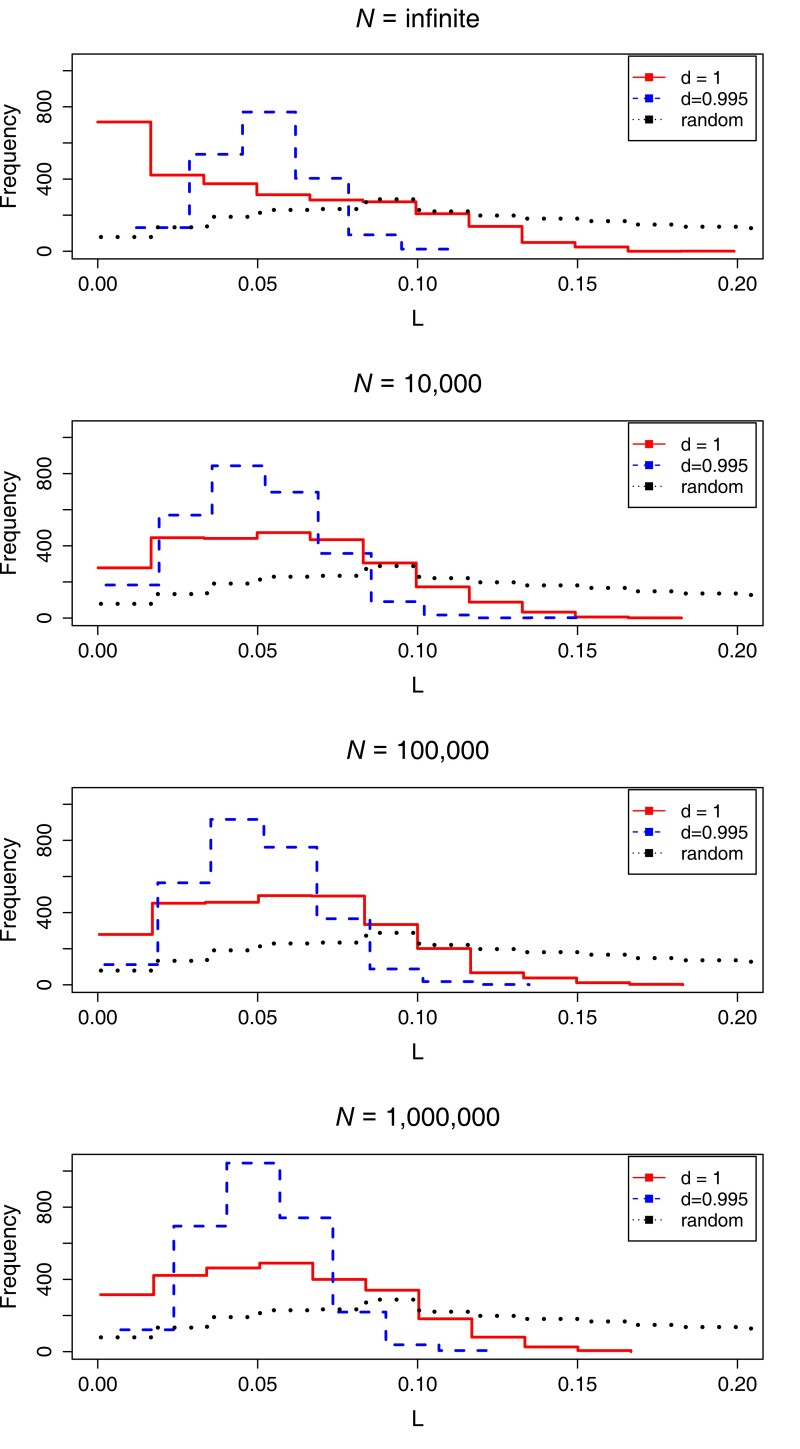
Distribution of *I* (the square of the distance from the centroid) for simulations resulting in 4 common alleles (*n_c_* = 4) for different environmental decay rates (*d*) and population sizes (*N*). The random expectation is shown as a black dotted line.

Figure 5 shows the distribution of *I* for *n_c_* = 4 for *d* = 1 and 0.995, compared with the null expectation, for infinite and finite (*N* = 10^4^) populations without generalized dominance. We see that in all cases, populations are more likely to have even common-allele frequencies (small values of *I*) than expected by chance. In other words, when we examine the common alleles in our simulated populations, their frequencies are more similar to each other than random values constrained to add to one would be.

Adding decay leads to more polymorphisms with almost even allele frequencies (*I* ∼ 0.05). Models with generalized dominance (Supplementary Fig. 9), however, produce less even allele frequencies. Populations with even allele frequencies are more resistant to the elimination of any alleles by genetic drift, since no allele is particularly rare.

## Discussion

### Monomorphisms and polymorphisms

Our simulations show that constructionist models of single-locus viability selection in a deteriorating environment can produce a wider range of numbers of alleles than found in previous studies. This scenario, in which the fitness of existing phenogenotypes incrementally decreases each generation, might be expected to produce higher levels of polymorphism: new mutations with higher fitnesses augment the existing allelic composition of the population, while the older alleles are being driven to extinction. But our results show that low levels of variation can also be generated.

For example, we can see (Fig. 2) that drawing mutational fitnesses from *U*[0, 1] with a decay parameter *d* of 0.999 (i.e. an across-the-board lowering of fitnesses by 0.1% each generation) led after 10,000 generations to populations with up to ∼40 alleles, of which up to 11 were common. These maxima compare to about half that number for total alleles (*n*), with the number of common alleles (*n_c_*) no more than 9 in populations under constant selection (Fig. 2). With the addition of drift due to a population size of *N* = 10,000, these ranges shrank, unsurprisingly, but the effect of the Red Queen remained, especially for the total numbers of alleles, between 1 and 18 for the Red Queen vs between 1 and 11 in her absence (Fig. 2).

The added realism of generalized dominance (which assumes that each allele has a primary effect that contributes to the viabilities of genotypes in which it is found) reinforces this finding. The same decay rate in an infinite population led to populations with values of *n* ranging from 1 to 41, and *n_c_* from 1 to 12; with drift in a population of 10,000, simulations gave up to *n* = 18 and up to *n_c_* = 8, respectively (Supplementary Fig. 4).

Our findings are important in attempts to explain the range of electrophoretic variants found in various studies. For example, Wellso *et al*. (1988) examined 15 presumptive electrophoretic loci in 2 wild populations of the Hessian Fly (*Mayetiola destructor*), finding all but one to be monomorphic. In contrast, Keith (1983) assayed large samples from 2 California populations of *Drosophila pseudoobscura* for variants of the esterase-5 protein, finding 22 and 33 different electromorphs, values that were minimum bounds for the numbers of alleles. A subsequent analysis of xanthine dehydrogenase proteins from these same populations implied 12 and 15 different alleles (Keith *et al*. 1985). Constructionist simulations to date have rarely generated allele numbers consistent with this range of variation (Spencer and Mitchell 2016).

It is worth noting that only a small rate of environmental decay is needed to significantly change the outcomes compared with those with constant selection parameters. For example, a decline of just one percent of 1% (*d* = 0.9999), an almost imperceptible decrease, changed the mean number of alleles at generation 10,000 by >50% (5.98–9.37).

### Models and assumptions

Many of the constructionist models are examples of what Servedio *et al.* (2014) call “proof-of-concept models,” designed to test a verbal hypothesis by examining the logical, mathematical consequences of its assumptions. In the case of constructionist models, the verbal hypothesis is simply that recurrent mutation and selection result in the buildup of genetic variation; the infinitesimal size of selection-coefficient parameter space affording polymorphism is easy to reach over time.

And, indeed, constructionist models of single-locus viability selection have shown that selection is more capable of maintaining higher levels of polymorphism than a naïve consideration of parameter-space sizes would suggest. This finding has proved robust to changes in the form of selection: almost always the temporal dimension of these models shows how adept an evolutionary process can be at seeking out unusual parts of parameter space.

Nevertheless, it is critical that even the high level of abstraction inherent in proof-of-concept models is underpinned by realistic assumptions. Most previous constructionist work has used constant fitnesses, an assumption that implies environmental constancy. Even the exceptions (models of frequency-dependent selection) have used parameters that varied only with allele and genotype frequencies. Hence, fitnesses in genetically identical populations at different times are unchanged. These assumptions imply that much of the selective environment has remained constant over thousands of generations.

The Red Queen hypothesis relaxes this assumption by assuming an ongoing deterioration of the fitnesses of extant types. Although biotic interactions are emphasized as the source of this decline in fitness, the Red Queen can also accommodate decay of the abiotic environment (Brockhurst *et al*. 2014). Our results reveal that previous constructionist findings about the ability to generate polymorphisms extend, at least qualitatively, to this new scenario. We note that this result was robust to whether the environmental decay each generation was constant or variable. Nevertheless, the temporally deteriorating fitnesses of the Red Queen can be investigated with only a historical approach and, hence, there is no parameter-space analysis with which to make a natural comparison.

Similarly, generalized dominance avoids the assumption that fitnesses of phenogenotypes that share alleles are independent. That each allele has some primary fitness consequences seems entirely reasonable. As in previous work on models with generalized dominance (Spencer and Mitchell 2016), we were able to generate monomorphisms in several of our runs. Thus, the incorporation of a more realistic fitness structure in a Red Queen setting has again resulted in theoretical outcomes that are closer to those found empirically.

These new simulations consistently generated populations with heterozygote advantage (Fig. 4 and Supplementary Fig. 8), as have all previous constructionist models and, indeed, other models using Fisher's geometric model of adaptation (Sellis *et al*. 2011). These findings, across a broad spectrum of assumptions, suggest that heterozygote advantage should be ubiquitous in natural populations. This expectation is in line with chemostat experiments using yeast as the model organism in which most novel mutations successfully invading the laboratory populations displayed heterozygote advantage in conjunction with other alleles present (Sellis *et al*. 2016; Aggeli *et al*. 2022).

A consideration of the requirement for a new mutation to invade an existing *n*-allele polymorphism explains this prediction. Invasion requires that the marginal fitness of the new mutation be greater than the population's mean fitness, and the former is most dependent on the *n* viabilities of the heterozygotes containing the mutation. The viability of the homozygous mutation is almost irrelevant due to the rarity of this phenogenotype. Mutations that have higher heterozygous viabilities are more likely to invade, thereby generating populations exhibiting heterozygote advantage. Hence, the paucity of good examples of heterozygote advantage in real populations (Hedrick 2012) is surprising and is poorly explained by our (and others') models. We take from this mismatch that the phenomenon of heterozygote advantage cannot arise at all easily in natural populations. It is so readily conserved by selection that any *de novo* cases must be exceptionally rare.

## Conclusion

In this paper, we model the outcome of natural selection and genetic drift acting on allelic variation in populations living in a gradually deteriorating environment and subject to recurrent mutation. The resultant levels of variation in our models are more in keeping with real data than the predictions of previous models have been. Hence, contrary to the claims of some writers, natural selection may be responsible for actively maintaining genetic variation in the wild.

## Supplementary Material

jkae107_Supplementary_Data

## Data Availability

The authors affirm that all data necessary for confirming the conclusions of the article are present within the article, figures, and Supplementary Material. Supplemental material available at G3 online.
