# Supplementary material for: Polymorphism and the Red Queen: the selective maintenance of allelic variation in a deteriorating environment
Source: G3 (Bethesda). 2024 May 21;14(7):jkae107. doi: 10.1093/g3journal/jkae107 (PMC11228834; doi:10.1093/g3journal/jkae107)
Supplement: jkae107_Supplementary_Data [file jkae107_supplementary_data.zip › File_S6_G3-2024-405115.pdf]

```
Program SandWManyGD;

  {$APPTYPE CONSOLE}

  {Many Runs of Spencer & Walter Simulation}

  uses
  SysUtils;

Const Maxgen = 10000;
      Maxallele = 200;
      MaxRun = 10000;
      Decay = 0.995;
      Alpha = 1.0/3.0;
      ExtThresh = 0.00005;  {Extinction threshold}


  Type BigArray = Array[1..Maxallele, 1..Maxallele] of Extended;

  Var N                     :Integer;
      Run                   :Integer;
      Wbar                  :Extended;
      SimpSeed, IP, JP               :Integer; {For Random Number Generation}
      C, CD, CM             :Extended;  {For Random Number Generation}
      Seed               :Array[1..4] of Integer;
      P                  :Array[1..Maxallele] of Extended;
      W                  :BigArray;  {Constants}
      X                  :Array[1..Maxallele] of Extended; {Primary Effect of Allele I}
      U                  :Array[1..97] of Extended;
      Outdata            :Text;  {Output file for statistical analysis}

  Function Uni: Extended;
    {Marsaglia et al. (1990) generator}

    Var Temp    :Extended;

    Begin
    Temp:=U[IP]-U[JP];
    If Temp<0.0 Then Temp:=Temp + 1.0;
    U[IP]:=Temp;
    IP:=IP-1;
    If IP=0 Then IP:=97;
    JP:=JP-1;
    If JP=0 Then JP:=97;
    C:=C-CD;
    If C<0.0 Then C:=C+CM;
    Temp:=Temp-C;
    If Temp<=0.0 Then Uni:=Temp + 1.0 Else Uni:=Temp
    End; {Of Function Uni}

  Procedure Randomize(IR, JR, KR, LR: Integer);
    Var II, JJ, MR    :Integer;
        S, T         :Extended;
    Begin
    For II:=1 To 97 Do
      Begin
      S:=0.0;
      T:=0.5;
      For JJ:=1 To 24 Do
       Begin
        MR:=(((IR*JR) MOD 179)*KR) MOD 179;
        IR:=JR;
       JR:=KR;
       KR:=MR;
       LR:=(53*LR + 1) MOD 169;
       If (LR*MR) MOD 64 >= 32 Then S:=S+T;
       T:=0.5*T
       End;
```

```
    U[II]:=S
    End;
  C:=362436.0/16777216.0;
  CD:=7654321.0/16777216.0;
  CM:=16777213.0/16777216.0;
  IP:=97;
  JP:=33
  End; {Of Procedure Randomize}

Procedure Startup;
  Var Filename      :String;

  Begin
  Writeln;
  Writeln;
  Writeln;
  Writeln('                Spencer & Marks Type Simulation for');
  Writeln;
  Writeln('     Red Queen Viability Selection Model with Generalized Dominance');
  Writeln;
  Writeln('           Hamish G. Spencer & Callum B. Walter January 2024');
  Writeln;
  Writeln;

  {Read in parameter values}
  Write('Enter random number seed: ');
  Readln(SimpSeed);
  Writeln;
  Seed[1]:= SimpSeed MOD 178 + 1;
  Seed[2]:= SimpSeed MOD 178 + 1;
  Seed[3]:= SimpSeed MOD 178 + 1;
  Seed[4]:= SimpSeed MOD 169;
  Randomize(Seed[1], Seed[2], Seed[3], Seed[4]);

  {Prepare Output file}
  Writeln('The output filenames will start with SW1GD');
  Write('Enter any further characters required in the name: ');
  Readln(Filename);
  Writeln;
  Filename:='SW1GD' + FloatToStr(Decay) + Filename + '.TXT';
  Assign(Outdata, Filename);
  Rewrite(Outdata)

  End; {Of Procedure Startup}

Procedure Mutation;

  Var I, Parent  :Integer;
      ParentThresh, SumFreq   : Extended;

  Begin
  ParentThresh := Uni;
  Parent := 0;
  SumFreq := 0.0;
  Repeat
    Parent := Parent + 1;
    SumFreq := SumFreq + P[Parent]
  Until SumFreq >= ParentThresh;
  {Parent is the existing allele that is going to mutate}
  If P[Parent] < ExtThresh Then
  {It is very rare and we need to ensure we don't get a negative P[N + 1]}
    Begin
    P[N + 1] := P[Parent];
    P[Parent] := 0.0
    End
  Else {P[Parent] >= ExtThresh}
    Begin
    P[N + 1] := ExtThresh;
```

```
    P[Parent] := P[Parent] - ExtThresh
    End;
  X[N+1] := Uni;
  For I:= 1 To N+1 Do
    Begin
    W[I, N+1] := Alpha*(X[I] + X[N+1]) + (1.0 - 2.0*Alpha)*Uni;
    W[N+1, I] := W[I, N+1]
    End;
  N := N+1
  End; {Of Procedure Mutation}

Procedure Selection;
  {Performs the changes in allele frequencies.}

  Var I, J, K              :Integer;
      TempMarg             :Extended;
      MargW                :Array[1..Maxallele] of Extended;

  Begin
  {First, calculate new marginal viabilities}
  For I:=1 to N Do
    Begin
    TempMarg:=0.0;
    For J:=1 To N Do TempMarg:=TempMarg + P[J]*W[I, J];
    MargW[I]:=TempMarg
    End;

  {Calculate new Wbar}
  Wbar:=0.0;
  For I:=1 To N Do Wbar:=Wbar + P[I]*MargW[I];

  {Calculate new P[I]s}
  For I:=1 To N Do P[I]:=P[I]*MargW[I]/Wbar;

  {Check for extinct alleles}
  K:=0;
  Repeat
    K:=K+1;
    If P[K] < ExtThresh Then
      Begin
      X[K]:=X[N];
      For I:=1 To N-1 Do
        Begin
        W[I,K]:=W[I,N];
        W[K,I]:=W[N,I]
        End;
      W[K,K] := W[N,N];
      P[K] := P[N];
      K := K-1; {Need to check if the new P[K] < extThresh}
      N := N-1
      End
  Until K >= N

  End; {Of Procedure Selection}

Procedure OneRun;

  Var Gen               :0..Maxgen;
      I, J, Nc              :Integer;
      L                     :Extended;
      SumHet, SumSqrHet     :Extended;
      SumHomo, SumSqrHomo   :Extended;
      MeanHet, VarHet, MeanHomo, VarHomo  :Extended;

  Begin
  {Set up Fitness matrix}
  X[1] := Uni;
  W[1,1] := (Alpha*2.0*X[1]) + ((1-2*Alpha)*Uni);
```

```
  N := 1;
  P[1] := 1.0;

  For Gen:=1 To MaxGen Do
    Begin
    Mutation;
    Selection;
    {Decay fitnesses}
    For I := 1 to N Do
      Begin
      X[I] := Decay*X[I];
      For J := 1 to N Do W[I,J] := Decay*W[I,J]
      End
    End;

  Nc := 0;
  For I := 1 to N Do if P[I] >= 0.01 Then Nc := Nc +1;

  L := 0.0;
  For I := 1 to N Do if P[I] >= 0.01 Then L := L + Sqr(P[I] - 1.0/Nc);

  Write(Outdata, Run:5, N:5, Nc:5, Wbar:10:4, L:10:4);

  {Calculate mean and variance of heterozygous and homozygous viabilities}
  SumHet := 0.0;
  SumSqrHet := 0.0;
  SumHomo := 0.0;
  SumSqrHomo := 0.0;
  For I := 1 To N Do
    Begin
    SumHomo := SumHomo + W[I,I];
    SumSqrHomo := SumSqrHomo +Sqr(W[I,I]);
    For J := I+1 to N Do
      Begin
      SumHet := SumHet + W[I,J];
      SumSqrHet := SumSqrHet +Sqr(W[I,J])
      End
    End;
  If N > 1 Then
    Begin
    MeanHet := SumHet/(N*(N-1)/2.0);
    VarHet := SumSqrHet/(N*(N-1)/2.0) - Sqr(MeanHet);
    Write(Outdata, MeanHet:10:4, VarHet:10:4)
    End
  Else Write(Outdata, '     .         .    ');
  MeanHomo := SumHomo/N;
  VarHomo := SumSqrHomo/N - Sqr(MeanHomo);
  Writeln(Outdata, MeanHomo:10:4, VarHomo:10:4)
  End; {Of Procedure OneRun}

Begin {******* Main Program ********}
Startup;
For Run:=1 To MaxRun Do OneRun;
Close(Outdata);
Writeln;
Writeln;
Writeln('Program successfully completed!');
Writeln;
Writeln('Hit any Enter key to continue');
Readln
End. {Of Program SandWManyGD}
```
