# Supplementary material for: Polymorphism and the Red Queen: the selective maintenance of allelic variation in a deteriorating environment
Source: G3 (Bethesda). 2024 May 21;14(7):jkae107. doi: 10.1093/g3journal/jkae107 (PMC11228834; doi:10.1093/g3journal/jkae107)
Supplement: jkae107_Supplementary_Data [file jkae107_supplementary_data.zip › Supplemental_Figure_Legends_G3-2024-405115.docx]

**Supplementary Figure Legends**

Fig. S1. Number of common alleles (*n_c_*), total number of alleles (*n*) and mean fitness ($\bar{\text{w}}$) over time in simulated populations with drift (population size *N* = 100,000, left column; *N* = 1,000,000, right column), for different environmental decay rates, *d*, as shown in each panel.

Fig. S2. Number of common alleles (*n_c_*), total number of alleles (*n*) and mean fitness ($\bar{\text{w}}$) over time in simulated populations without drift. The left-hand column graphs are for variable environmental decay rates, which were sampled from Norm(*d*, (1 – *d*)^2^), as shown in each panel, which produced 15.87% of values > 1. The right-hand column pertains to the mutational model of generalized dominance, with *α* = ⅓.

Fig. S3. Mean number of total alleles (*n*, solid lines) and common alleles (*n_c_*, dashed lines) for different decay rates (indicated by the different colours) as a function of different population sizes. The rightmost points are for an infinite population with variable decay, with *d* sampled from Norm(*d*, (1 – *d*)^2^).

Fig. S4. Frequency distributions of the numbers of common (*n_c_*) and total (*n*) alleles at generation 10,000 in from 10^4^ simulations with drift (population size *N* = 100,000, left column; *N* = 1,000,000, right column). Means of each distribution are shown in each panel.

Fig. S5. Frequency distributions of the numbers of common (*n_c_*) and total (*n*) alleles at generation 10,000 in from 10^4^ simulations with variable decay, with *d* sampled from Norm(*d*, (1 – *d*)^2^). Means of each distribution are shown in each panel.

Fig. S6. Frequency distributions of the numbers of common (*n_c_*) and total (*n*) alleles at generation 10,000 in from 10^4^ simulations with generalized dominance with (*α* = ⅓) without drift (left column) and with drift (*N* = 10,000, right column). Means of each distribution are shown in each panel.

Fig. S7. Distributions of the mean fitness ($\bar{\text{w}}$) at generation 10,000 in 10^4^ simulations with drift (*N* = 10,000 and *N* = 1,000,000, as shown), for the final total numbers of alleles (*n*; lefthand column) and common alleles (*n_c_*; righthand column). Means of each distribution are shown in red.

Fig. S8. Scatterplots of the mean of the homozygote viabilities versus the mean of the heterozygote viabilities for 10^4^ simulations each at generation 10,000, with drift (*N* = 100,000, lefthand column; *N* = 1,000,000, righthand column), for different decay rates as shown in the panels. Pearson’s correlation values are shown in each panel.

Fig. S9. Distribution of *I* (the square of the distance from the centroid) for generalized-dominance simulations resulting in 4 common alleles (*n_c_* = 4) for different environmental decay rates (*d*) and population sizes (*N*). The random expectation is shown as a black dotted line.
