# Supplementary material for: Polymorphism and the Red Queen: the selective maintenance of allelic variation in a deteriorating environment
Source: G3 (Bethesda). 2024 May 21;14(7):jkae107. doi: 10.1093/g3journal/jkae107 (PMC11228834; doi:10.1093/g3journal/jkae107)
Supplement: jkae107_Supplementary_Data [file jkae107_supplementary_data.zip › Supplemental_File_Legends_G3-2024-405115.docx]

**Supplementary Files Descriptions**

The following files contain Delphi code for the various computer programs described and used in this paper.

**File S1: Program SandWSingle**: Code for a single run of the model with constant *d*, mutational fitness drawn independently from U[0, 1] and no genetic drift. Total and common allele numbers and population mean fitness are recorded every generation.

**File S2: Program SandWSingleDrift**: Code for a single run of the model with constant *d*, mutational fitness drawn independently from U[0, 1] and genetic drift. Total and common allele numbers and population mean fitness are recorded every generation.

**File S3: Program SandWSingleGD**: Code for a single run of the model with constant *d*, mutational fitness generated according to the model of generalized dominance (see text) and no genetic drift. Total and common allele numbers and population mean fitness are recorded every generation.

**File S4: Program SandWMany**: Code for many runs of the model with constant *d*, mutational fitness drawn independently from U[0, 1] and no genetic drift. Total and common allele numbers, allele frequencies, fitness properties and population mean fitness are recorded for each run at Generation 10,000.

**File S5: Program SandWManyDrift**: Code for many runs of the model with constant *d*, mutational fitness drawn independently from U[0, 1] and genetic drift. Total and common allele numbers, allele frequencies, fitness properties and population mean fitness are recorded for each run at Generation 10,000.

**File S6: Program SandWManyGD**: Code for many runs of the model with constant *d*, mutational fitness generated according to the model of generalized dominance (see text) and no genetic drift. Total and common allele numbers, allele frequencies, fitness properties and population mean fitness are recorded for each run at Generation 10,000.

**File S7: Program SandWManyDriftGD**: Code for many runs of the model with constant *d*, mutational fitness generated according to the model of generalized dominance (see text) and genetic drift. Total and common allele numbers, allele frequencies, fitness properties and population mean fitness are recorded for each run at Generation 10,000.

**File S8: Program SandWVarSingle**: Code for a single run of the model with randomly distributed *d* values, mutational fitness drawn independently from U[0, 1] and no genetic drift. Total and common allele numbers and population mean fitness are recorded every generation.

**File S9: Program SandWVarMany**: Code for many runs of the model with randomly distributed *d* values, mutational fitness drawn independently from U[0, 1] and no genetic drift. Total and common allele numbers, allele frequencies, fitness properties and population mean fitness are recorded for each run at Generation 10,000.
