# Supplementary figures and images for: Polymorphism and the Red Queen: the selective maintenance of allelic variation in a deteriorating environment
Source: G3 (Bethesda). 2024 May 21;14(7):jkae107. doi: 10.1093/g3journal/jkae107 (PMC11228834; doi:10.1093/g3journal/jkae107)

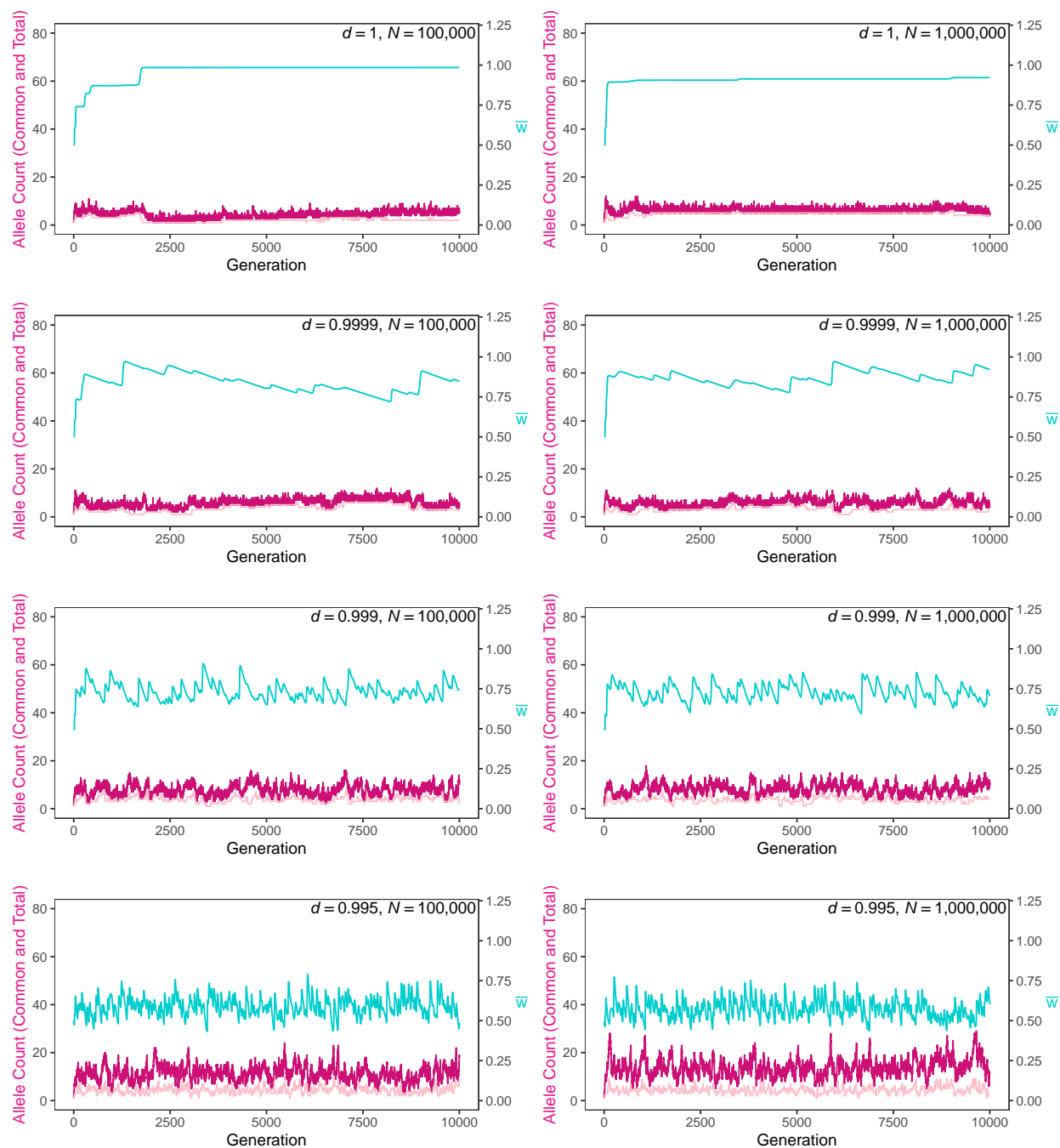

Fig S1

Supplement: jkae107_Supplementary_Data [file jkae107_supplementary_data.zip › Fig._S1_G3-2024-405115.pdf]

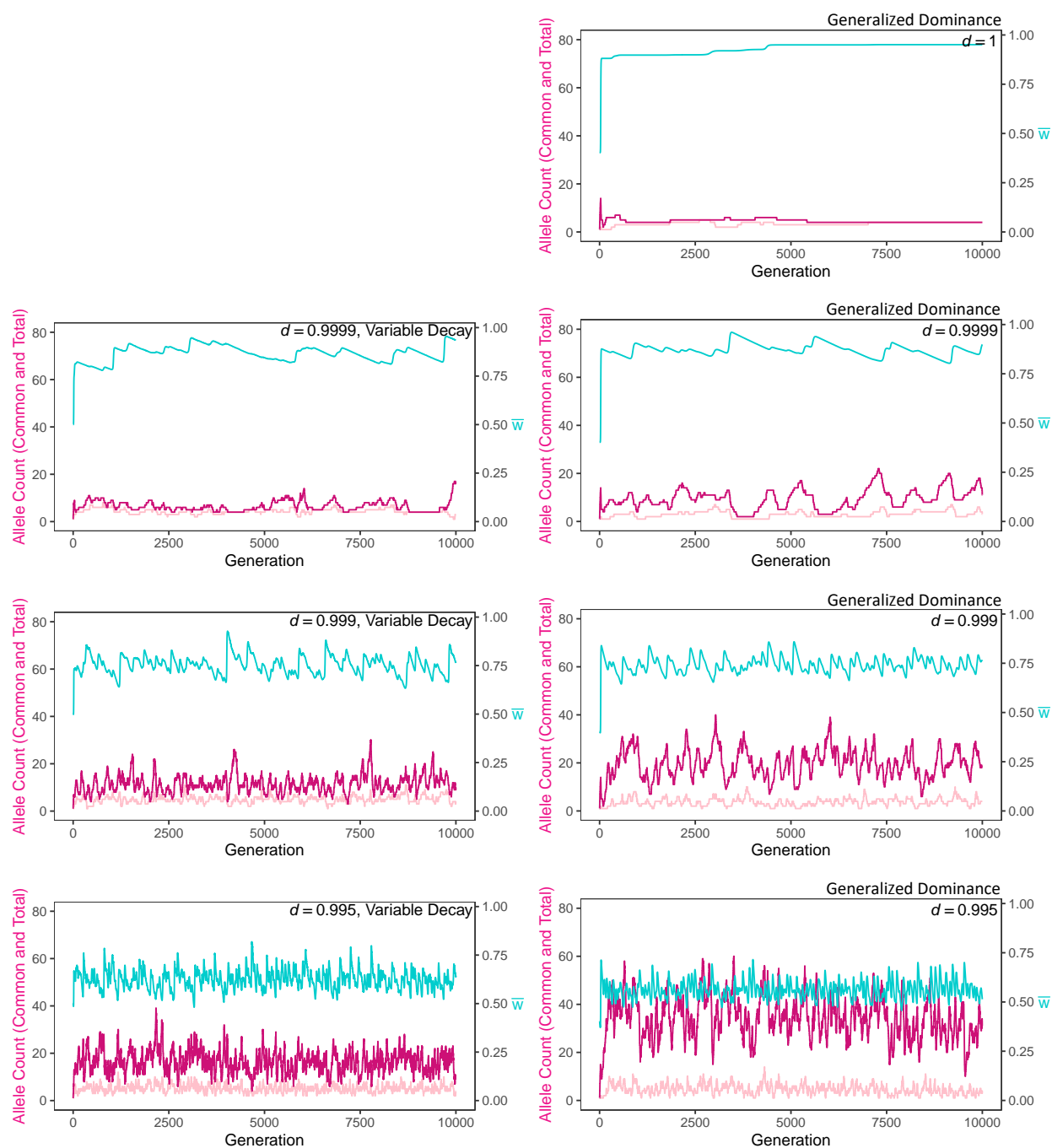

Fig S2

Supplement: jkae107_Supplementary_Data [file jkae107_supplementary_data.zip › Fig._S2_G3-2024-405115.pdf]

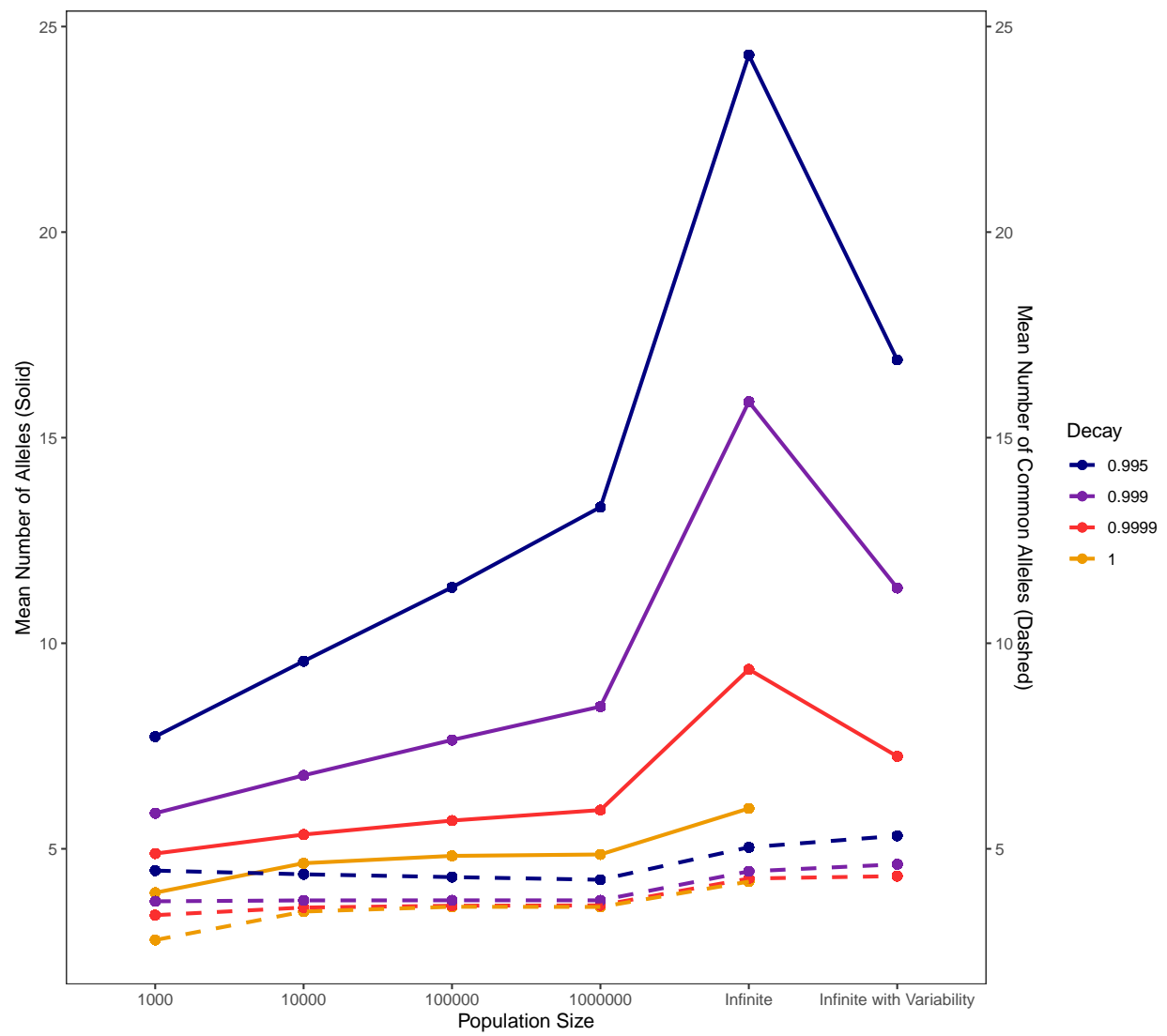

Fig S3

Supplement: jkae107_Supplementary_Data [file jkae107_supplementary_data.zip › Fig._S3_G3-2024-405115.pdf]

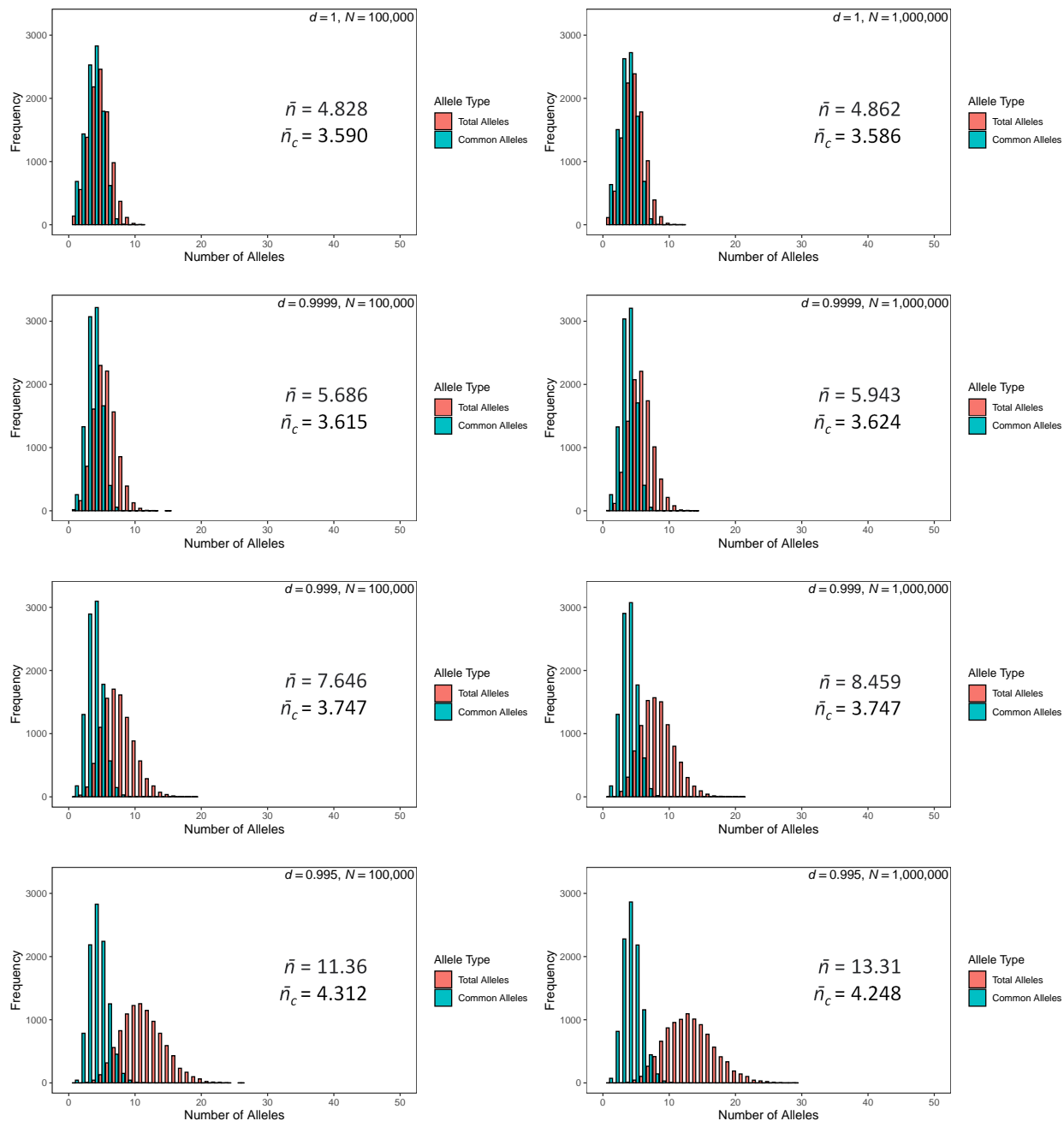

Fig S4

Supplement: jkae107_Supplementary_Data [file jkae107_supplementary_data.zip › Fig._S4_G3-2024-405115.pdf]

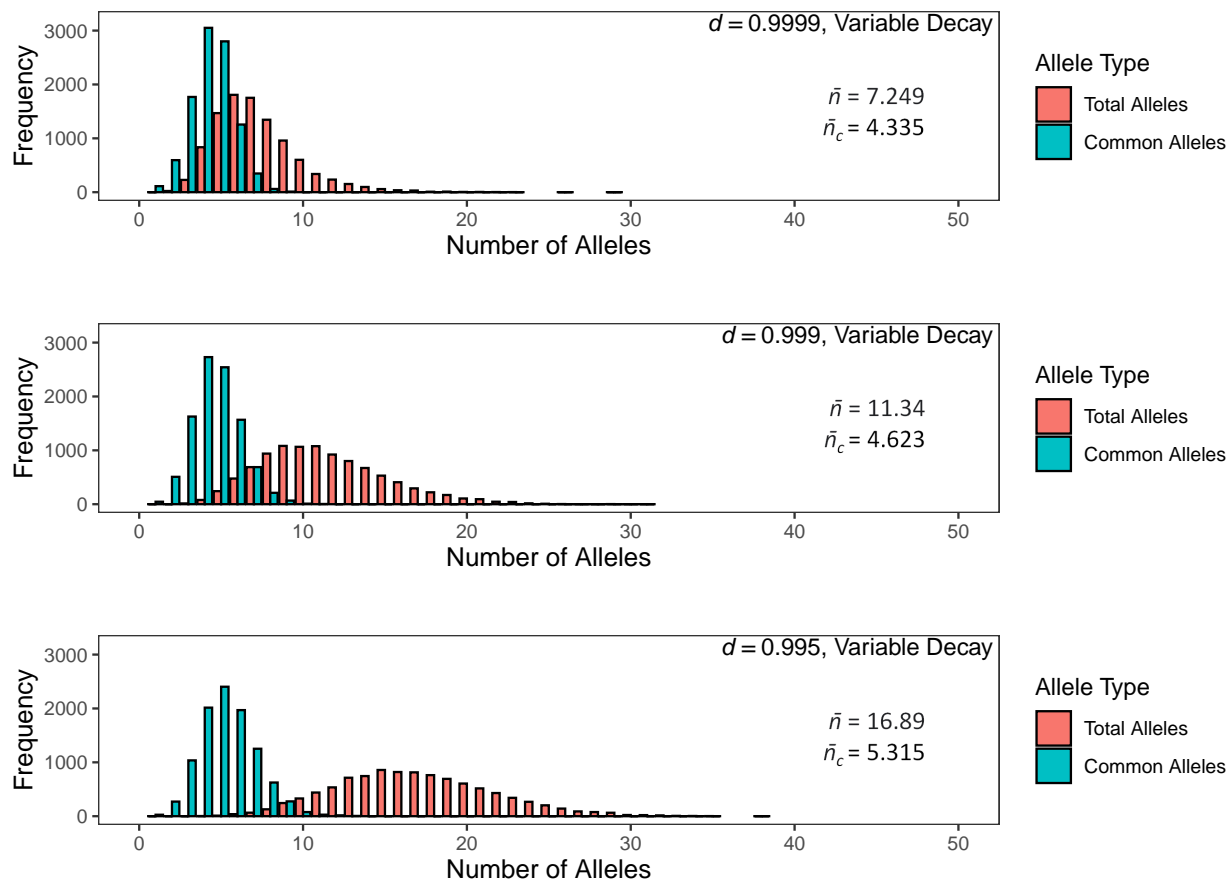

Fig S5

Supplement: jkae107_Supplementary_Data [file jkae107_supplementary_data.zip › Fig._S5_G3-2024-405115.pdf]

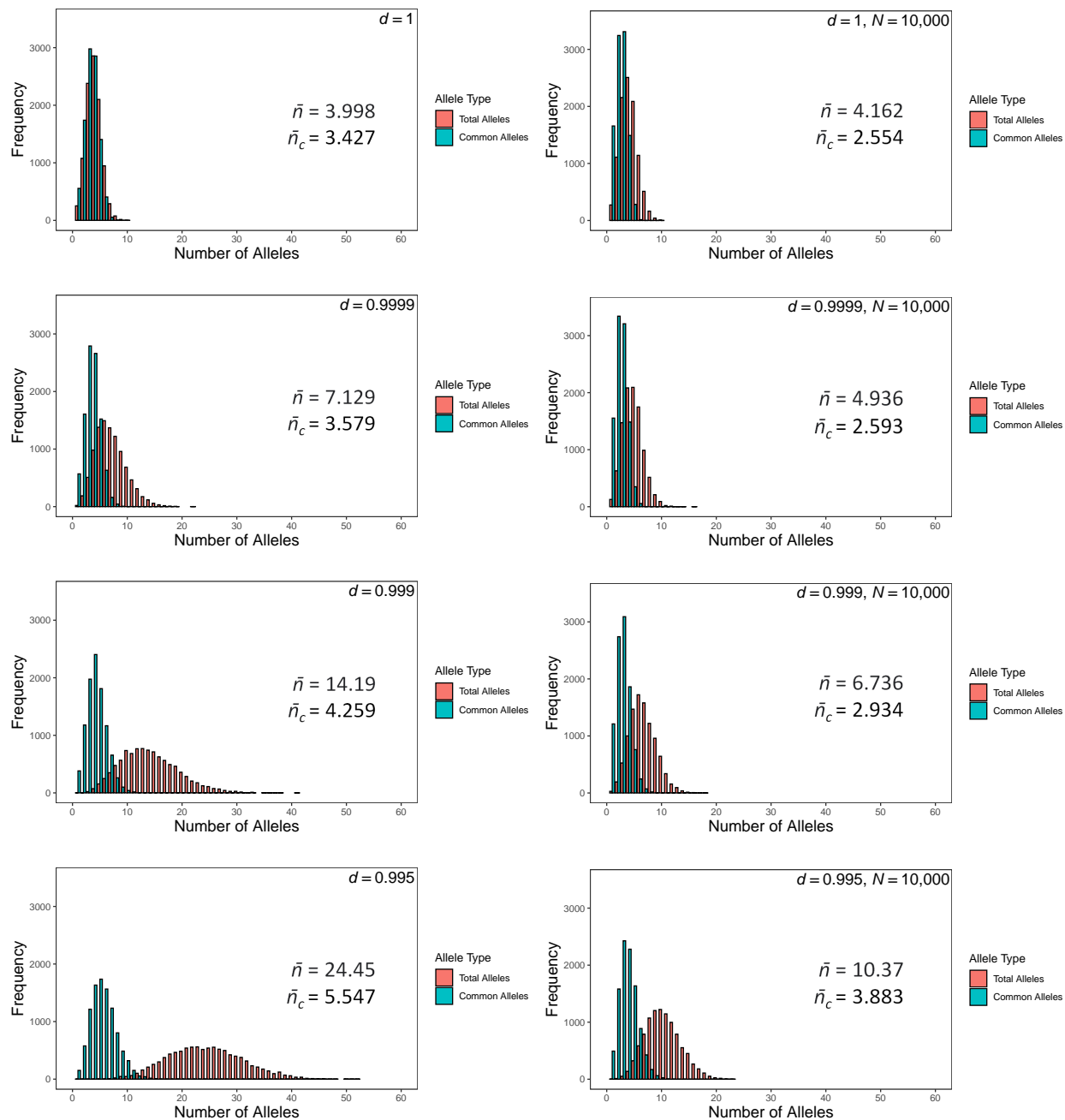

Fig S6

Supplement: jkae107_Supplementary_Data [file jkae107_supplementary_data.zip › Fig._S6_G3-2024-405115.pdf]

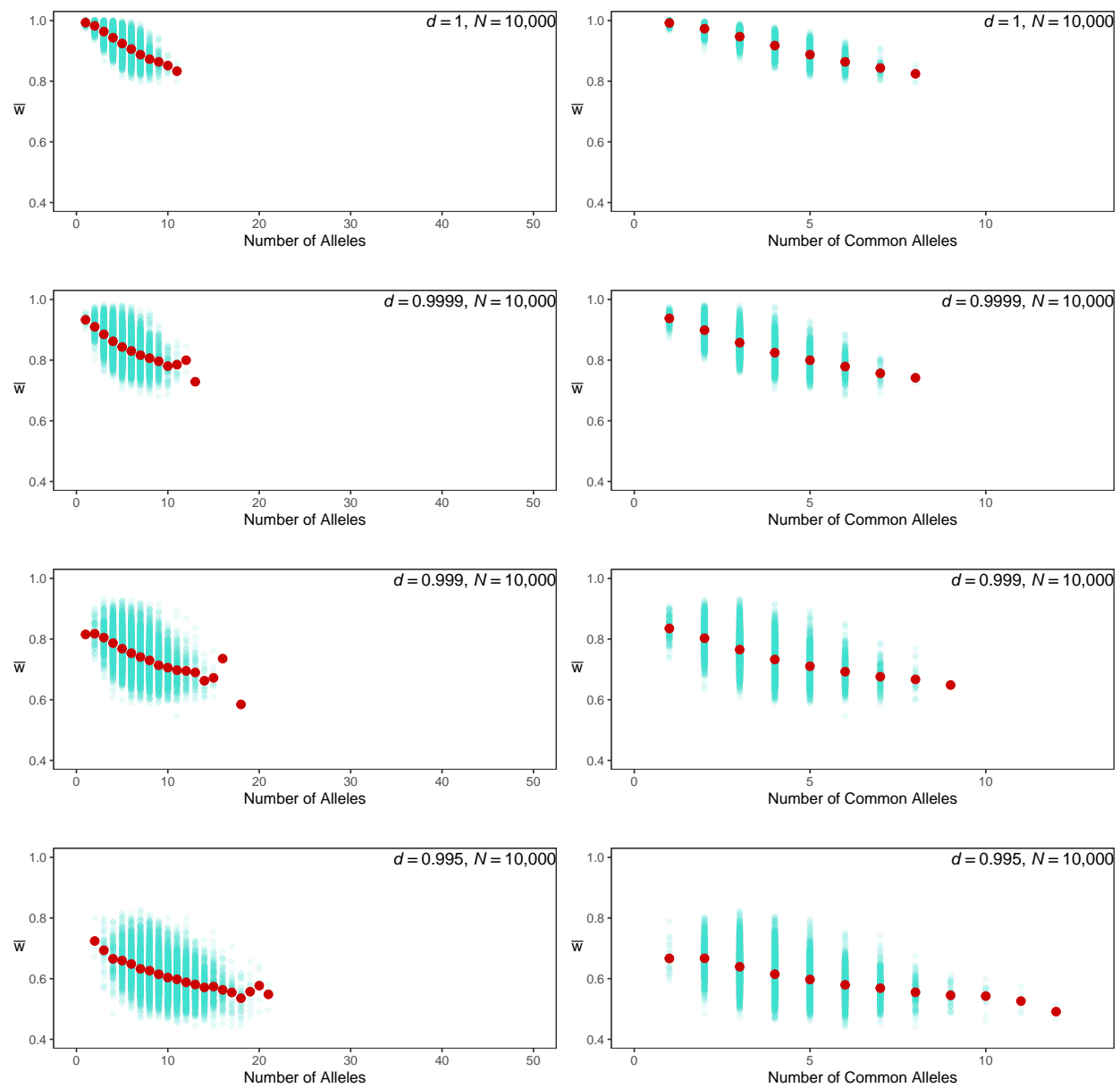

Fig S7p1

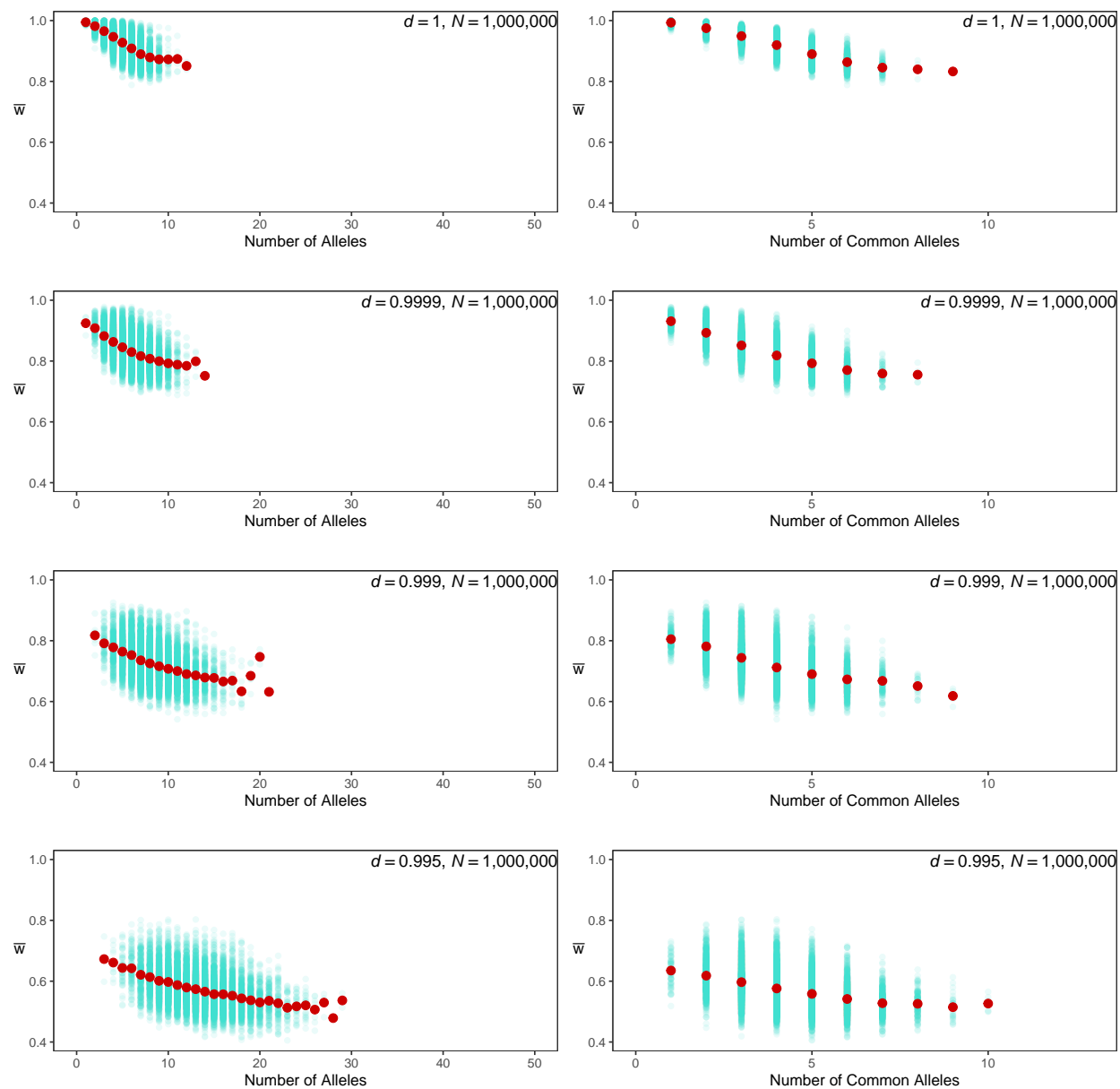

Fig S7p2

Supplement: jkae107_Supplementary_Data [file jkae107_supplementary_data.zip › Fig._S7_G3-2024-405115.pdf]

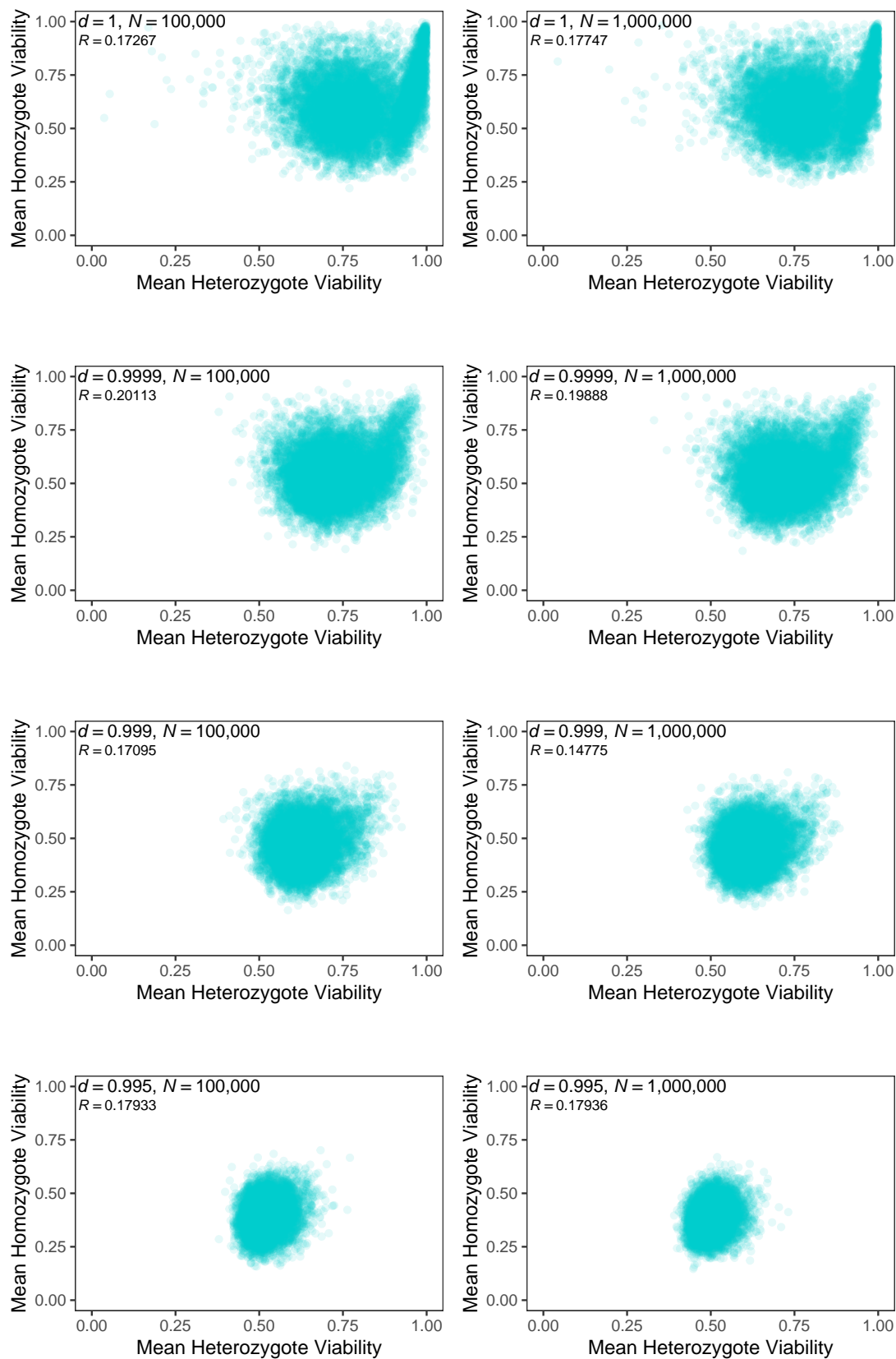

Fig S8

Supplement: jkae107_Supplementary_Data [file jkae107_supplementary_data.zip › Fig._S8_G3-2024-405115.pdf]

### $N = \text{Infinite}$ , Generalized Dominance

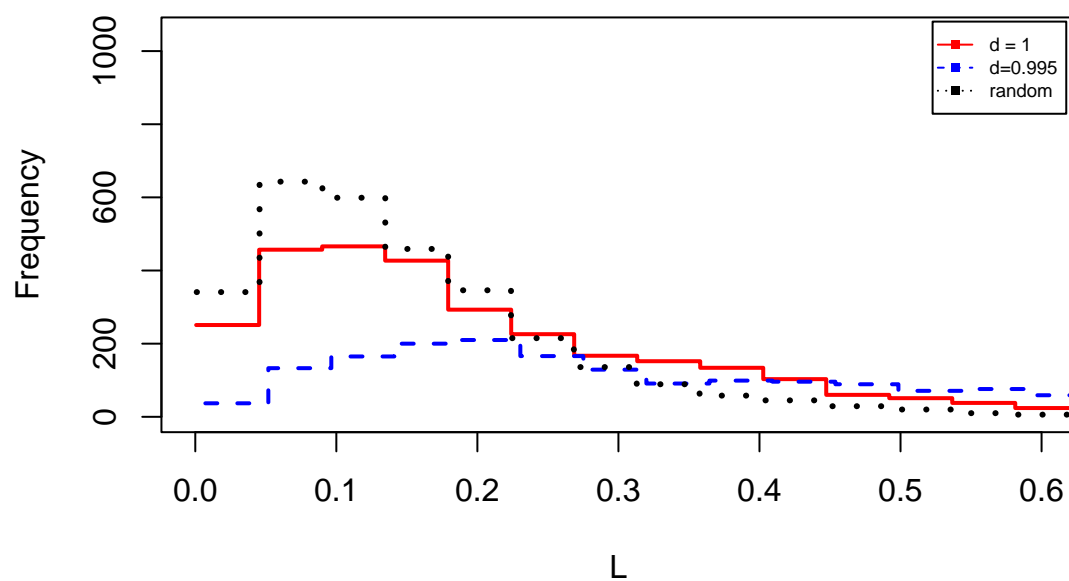

### $N = 10,000$ , Generalized Dominance

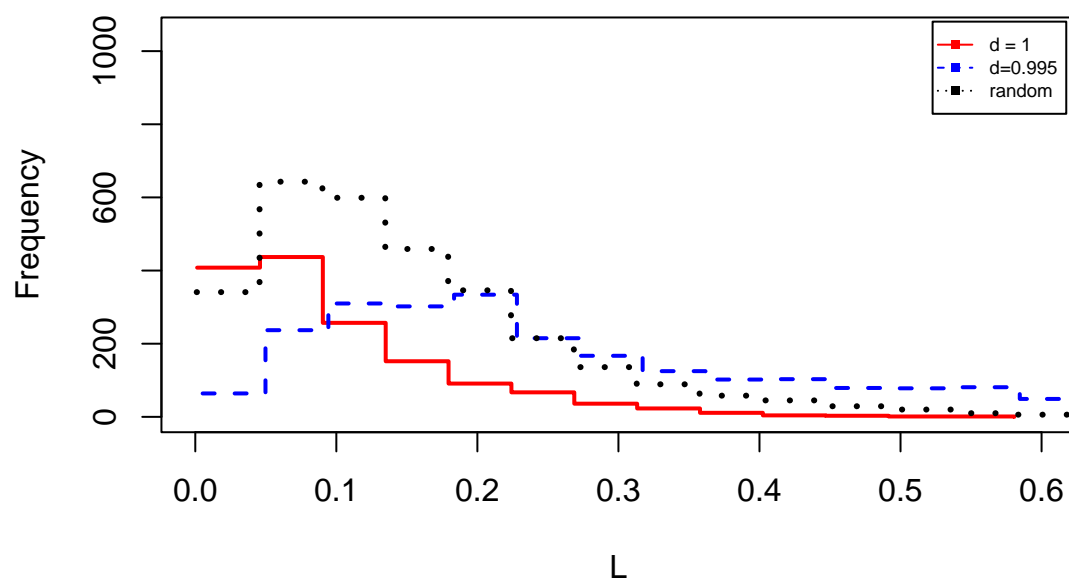

Fig S9

Supplement: jkae107_Supplementary_Data [file jkae107_supplementary_data.zip › Fig._S9_G3-2024-405115.pdf]
